# Rheological Properties and Influence Mechanisms of Twin-Screw Activated Rubber Powder Composite SBS-Modified Asphalt

**DOI:** 10.3390/ma18102359

**Published:** 2025-05-19

**Authors:** Yicai Zhao, Rui Dong, Jingzhuo Zhao, Yongning Wang, Fucheng Guo, Xiaolong Wei, Bo Li, Yong Huang

**Affiliations:** 1 Gansu Transportation Investment Management Co., Ltd., Lanzhou 730030, China; fhmacd@163.com; 2 Gansu Provincial Transportation Planning Survey and Design Institute Co., Ltd., Lanzhou 730030, China; 18993250394@139.com (R.D.); 13679424751@163.com (Y.W.); wei-xl@163.com (X.W.); 3 School of Chemistry, Xinjiang University, Boda Campus, Urumqi 830046, China; pengyou0991@163.com; 4 School of Civil Engineering, Lanzhou Jiaotong University, Lanzhou 730070, China; libolzjtu@hotmail.com

**Keywords:** road engineering, twin-screw extruder, activated rubber composite-modified asphalt, rheological properties, microscopic influence mechanism

## Abstract

To investigate the rheological properties and influence mechanisms of twin-screw activated rubber composite-modified asphalt, we used SBS-modified asphalt (SBS) as the reference. Raw rubber powder composite-modified asphalt (RA/SBS) and activated rubber composite-modified asphalt (ARA/SBS) were prepared. A dynamic shear rheometer (DSR) and bending beam rheometer (BBR) were employed to comparatively analyze the rheological characteristics of the three modified asphalts, while Fourier transform infrared spectroscopy (FTIR) and fluorescence microscopy were used to reveal the micro-mechanisms in ARA/SBS. The results showed that ARA/SBS exhibited better storage stability and low-temperature flexibility compared to SBS and RA/SBS, and ARA/SBS demonstrated lower viscosity than RA/SBS. Among the three, ARA/SBS showed significantly improved high-temperature performance. The comparison of creep stiffness S and creep rate m indicated optimal performance in ARA/SBS, confirming that twin-screw activated rubber powder could significantly enhance the low-temperature properties of modified asphalt. Microscopically, chemical reactions occurred between oxygen-containing functional groups in activated rubber and polar groups in asphalt, while a cross-linked network structure formed between activated rubber molecules and asphalt molecular chains, improving compatibility and enhancing the rheological properties of composite modified asphalt.

## 1. Introduction

In recent years, asphalt pavement has been widely used in road construction and maintenance projects due to its excellent road performance. However, conventional asphalt materials cannot meet the demands of social development. Through prolonged research and exploration, it has been found that crumb rubber-modified asphalt (CRMA), produced by adding waste tire rubber powder to asphalt, exhibits superior high-temperature ductility and low-temperature crack resistance. This material also helps alleviate “black pollution” issues [1,2,3]. Consequently, waste rubber has gained extensive application in highway engineering and has become one of the hotspot materials for future research in this field.

However, waste tire crumb rubber powder primarily consists of vulcanized rubber with a three-dimensional cross-linked network structure between molecular chains. This unique topological configuration creates significant steric hindrance between rubber particles and the asphalt matrix, preventing effective molecular-level integration. Consequently, crumb rubber-modified asphalt suffers from three major issues: poor storage stability, high viscosity, and malodorous emissions [3,4]. To address this, researchers primarily employ activation methods for rubber powder pretreatment, including physical, chemical, and biological activation. Among these, twin-screw activation proves more amenable to industrial application [4,5,6]. Twin-screw technology enables the activation processing of waste tire rubber powder under coupled mechanical, thermal, and chemical energy inputs without damaging the main rubber chain structure. The synergistic effects of high temperature, mechanical energy, and chemical energy break the internal cross-linked network system of rubber powder, achieving deep activation while preserving material performance. Compared to static mixing or single-screw processes, twin-screw activation demonstrates over 20% higher efficiency with superior activation performance [7].

In the study of the performance characterization and influencing mechanisms of activated rubber powder-modified asphalt, Jin et al. found that the main reason for the incompatibility between rubber powder and base asphalt lies in the significant differences in chemical properties and physical characteristics between them [5]. Li et al. discovered that activating rubber powder with NaClO solution results in a rougher surface morphology and enhances its integration with asphalt [8], while Shatanawi et al. also confirmed that activated rubber powder improves compatibility with asphalt [9]. Tao et al. demonstrated that microwave treatment enhances the rutting resistance and aging resistance of rubberized asphalt [10], which was further validated by Sousa et al. [11]. Chen et al. reported that activated rubber powder significantly improves the high-temperature, fatigue, and low-temperature performance of asphalt [12], though Ying et al. observed varying degrees of fatigue performance degradation in rubber-modified asphalt depending on the activation methods [13]. Sheng et al. revealed through DSR and BBR tests that activated rubber powder enhances asphalt’s resistance to permanent deformation [14]. Hosseinnezhad et al. discovered that at the microscopic level, activated rubber powder increases asphalt’s fracture energy, thereby improving the road performance of rubber-modified asphalt [15]. Zhu et al. identified new benzene ring structures and the breaking of C-C and C-S bonds with partial C=C bond formation during rubber powder activation [16]. In summary, rubber powder desulfurization can improve the road performance of rubber-modified asphalt to varying degrees, but the road performance mechanisms of activated rubber powder compounded with SBS-modified asphalt remain unclear.

Therefore, this study prepared ARA/SBS using twin-screw activated rubber powder, with SBS and RA/SBS as references. DSR and BBR tests were employed to investigate the rheological properties’ variation patterns in ARA/SBS, SBS, and RA/SBS. The influence mechanism of twin-screw activated rubber powder on the rheological performance of rubber powder composite SBS-modified asphalt was analyzed through infrared spectroscopy and fluorescence microscopy tests, aiming to establish solid technical support for large-scale applications of activated rubber powder.

## 2. Raw Materials and Test Methods

### 2.1. Raw Materials

The base asphalt used was Zhenhai base asphalt with a penetration grade of 90. The rubber powder was 40-mesh rubber powder produced by Wuwei Xinhaoyuan Environmental Technology Co., Ltd. in Wuwei, China. The SBS modifier was 1301 linear modifier produced by Dushanzi Petrochemical Company in Karamay, China. The compatibilizer uniformly disperses modifiers in asphalt through high-speed shearing and stirring, enhancing compatibility between rubber powder and base asphalt. The stabilizer inhibits phase separation between rubber powder and base asphalt during storage, ensuring long-term stable performance. Both components synergistically ensure the modified asphalt’s high-temperature deformation resistance and low-temperature crack resistance, with compatibilizer and stabilizer provided by Gansu Provincial Transportation Planning Survey & Design Institute Co., Ltd. (Lanzhou, China). The conventional performance test results of Zhenhai 90# base asphalt are shown in Table 1; the physicochemical technical indicators of the 40-mesh rubber powder are listed in Table 2. The main properties of the furfural extract oil are presented in Table 3. The quality parameters of the stabilizer are detailed in Table 4.

### 2.2. Sample Preparation

(1) Activated Rubber Powder Preparation

**Figure 1:**
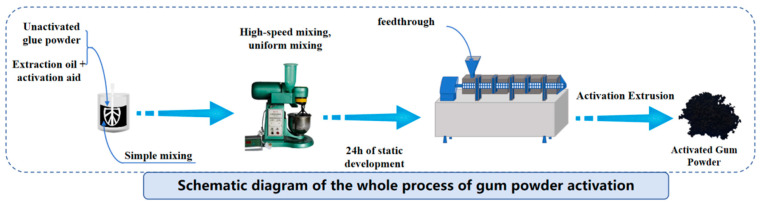
Schematic diagram of the preparation process of twin-screw activated rubber.

The activated rubber powder was prepared using a large-scale twin-screw extrusion equipment produced by Yenching Asphalt Technology Co., Ltd., Xi’an, China. The specific activation process is as follows: (1) pretreatment of activator and extracted oil: heated in a 75 °C oven for 1 h to reach Newtonian fluid state before use, (2) thoroughly mix the activator with extracted oil, and then add the mixture to rubber powder and homogenize using a mixer, (3) let the uniformly mixed rubber powder rest at room temperature for 12 h for full development, (4) feed the developed rubber powder into twin-screw extrusion equipment for activation granulation. The preparation process of activated rubber particles is shown in Figure 1.

In this paper, an anisotropic meshing class twin-screw extruder was used, consisting of six temperature-controlled sections, with the temperatures set to 240 °C in the conveying section, 260 °C in the melting section, 280 °C in the mixing section, 260 °C in the exhausting section, 240 °C in the homogenizing section, and 150 °C in the head, with a screw speed of 80 r/min, feed rate of 25 kg/h, and pressure at the end of the machine of 23 bar.

(2) Preparation of Activated Rubber Powder Composite SBS-Modified Asphalt

**Figure 2:**
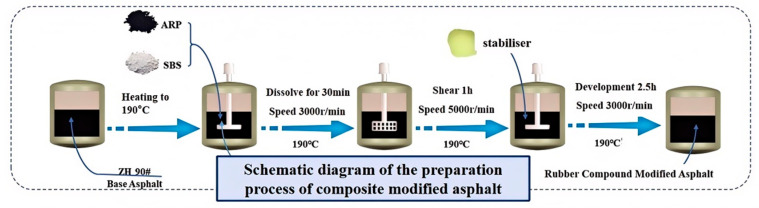
Modified asphalt preparation process.

The rubber composite-modified asphalt was prepared using the integrated asphalt swelling and shearing machine from Gansu Provincial Transportation Planning, Survey & Design Institute Co., Ltd. (Lanzhou, China). The specific preparation process was as follows: (1) Rapidly heat base asphalt to 163 °C, and then add furfural extract oil, activated rubber particles, and SBS modifier. Raise the temperature to 190 °C for swelling (3000 rpm for 30 min). (2) Shear for 1 h at 5000 rpm. (3) Add stabilizer and develop for 2.5 h at 3000 rpm. The preparation process is shown in Figure 2. RA/SBS, SBS, and ARA/SBS asphalts were prepared using the same process (for SBS preparation, the temperature was reduced to 175 °C to avoid performance degradation of the modified asphalt from excessive temperatures). In this preparation process, the temperature and shear speed must be strictly controlled. An excessively low temperature results in overly high viscosity, preventing full contact between the rubber powder and asphalt and compromising their compatibility. Shear speed directly affects the particle size and dispersion of the rubber powder in asphalt. Insufficient speed reduces the stability of the entire asphalt system, negatively impacting properties such as softening point, ductility, and viscosity [10,15].

During the preparation processes of the three types of asphalt mentioned above, the same dosage of SBS modifier was added. Equal amounts of activated rubber particles and raw rubber powder were incorporated into ARA/SBS and RA/SBS, respectively. Using SBS-modified asphalt as a reference, comparisons between ARA/SBS and RA/SBS were conducted to investigate their conventional performance, rheological characteristics, and microscopic mechanisms.

### 2.3. Test Method

(1) Routine Performance Test

According to the specifications in “Test Methods of Bitumen and Bituminous Mixtures for Highway Engineering” (JTG E20-2011) [17], the conventional performance indicators including penetration, softening point, ductility, viscosity, and segregation of the three asphalt types were tested. The relevant data were analyzed to derive evaluations and principles regarding their high- and low-temperature performance.

(2) Infrared Spectroscopy Testing

**Figure 3:**
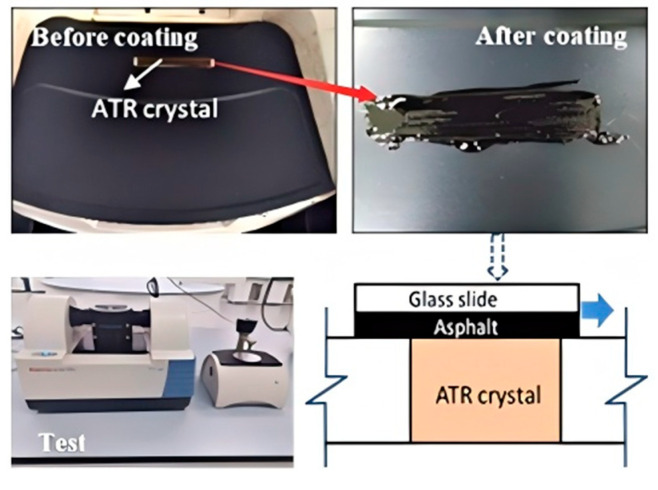
Infrared spectroscopy experiment.

This study utilized a Thermo Nicolet infrared spectrometer (Waltham, MA, USA) to obtain infrared spectra of three asphalt types. By analyzing the infrared light absorption characteristics of the materials, specific chemical bonds and functional group information in their molecular structures were revealed. The key experimental parameters included a scanning range of 400 cm^−1^ to 4000 cm^−1^, minimum resolution of 0.019 cm^−1^, and 32 scans. The experimental testing process is illustrated in Figure 3.

(3) Dynamic Shear Rheological Test

**Figure 4:**
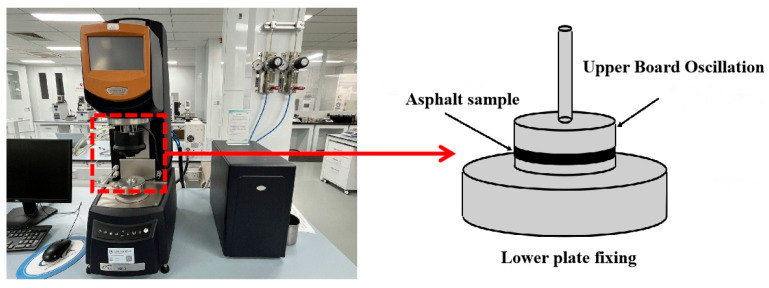
Dynamic shear rheological test.

This study employed the HR10 dynamic shear rheometer produced by TA Instruments, New Castle, DE, USA, to conduct dynamic shear tests on three modified asphalt types. A comprehensive analysis was performed on their high-temperature performance and fatigue characteristics, with test temperatures ranging from 58 °C to 82 °C and a fixed frequency of 10 rad/s. For the MSCR test, a 25 mm rotor was selected with a 1000 μm gap setting between the rotor and DSR’s lower parallel plate, as shown in Figure 4, illustrating the experimental procedure.

(4) Bending Beam Rheological Test

**Figure 5:**
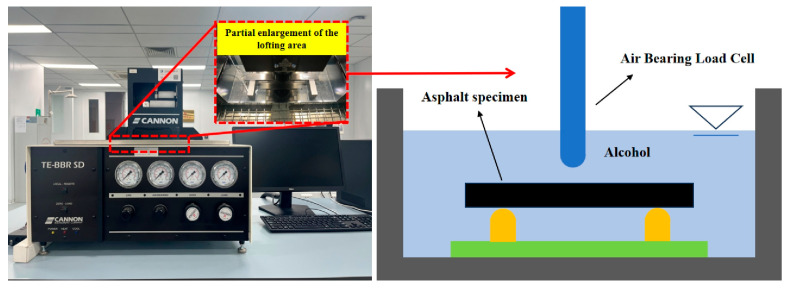
Bending beam rheological test.

This study employs a TE-BBR SD bending beam rheometer, with BBR test temperatures set at 10 °C above the pavement’s lowest temperature. According to the time–temperature superposition principle for asphalt materials, the loading duration can be reduced to 60 s. The 60 s stiffness modulus obtained from the test essentially equates to the 2 h stiffness modulus at the minimum design temperature. Three test temperatures were established: −12 °C, −18 °C, and −24 °C. The experimental procedure is illustrated in Figure 5.

(5) Fluorescence microscope experiment

**Figure 6:**
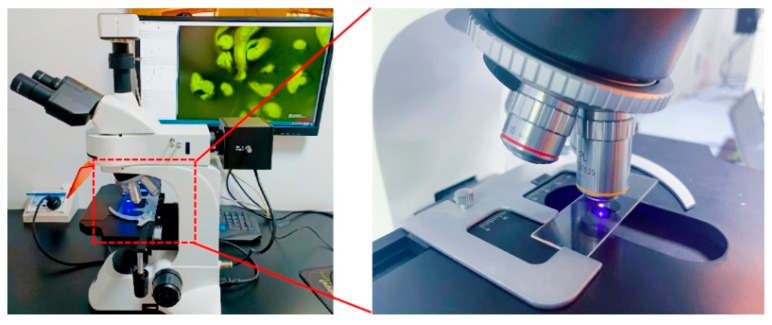
Fluorescence observation experiment.

Certain components in asphalt (such as aromatic hydrocarbons, the styrene segment of the polymer modifier SBS) emit fluorescence when excited by a specific light wavelength (e.g., ultraviolet or blue light). The compatibility differences between polymers and the asphalt matrix in modified asphalt lead to phase separation, where fluorescence signal variations can distinguish the two phases. This study employed a DFM-66C (made in China Caikang Opticai Instruments Co., Shanghai, China) fluorescence microscope to observe the microscopic morphology and material dispersion in asphalt. The microstructures of three modified asphalt samples were examined using 100× magnification fluorescence microscopy, as shown in Figure 6.

## 3. Experimental Results and Analysis

In the preparation process of the three asphalt types studied in this paper, an identical dosage of SBS modifier was incorporated. Both ARA/SBS and RA/SBS contained equivalent amounts of activated rubber powder and raw rubber powder, respectively. Using SBS-modified asphalt as the reference, comparisons were made between ARA/SBS and RA/SBS to investigate their conventional properties, rheological characteristics, and influencing mechanisms. All experiments mentioned were verified through three sets of reproducibility tests, with sample variations within ±3% error margins, ensuring the authenticity and reliability of the experimental data.

### 3.1. Conventional Performance Testing Analysis

**Figure 7:**
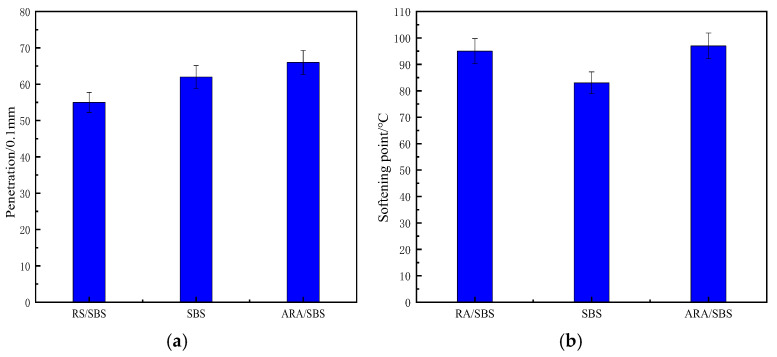

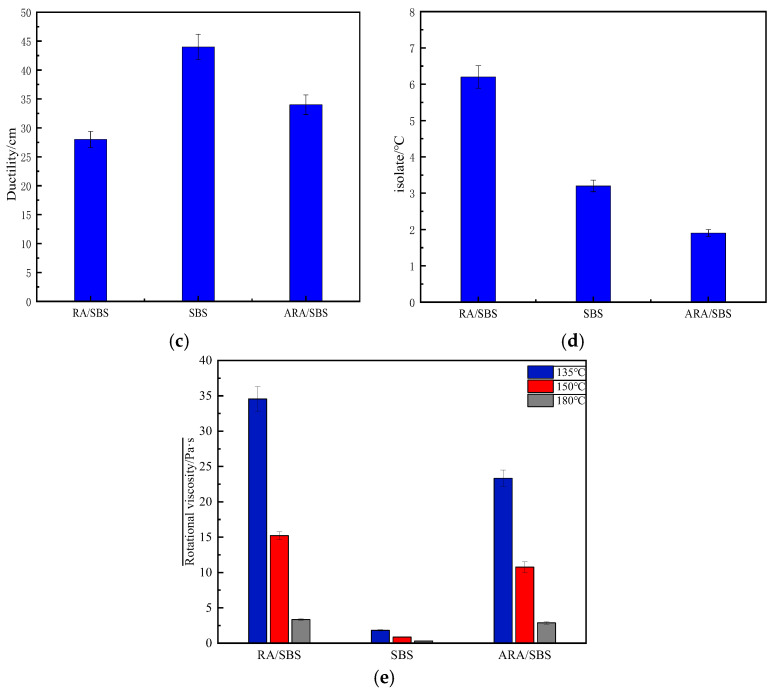
Routine performance testing. (**a**) Penetration; (**b**) softening point; (**c**) ductility; (**d**) isolate; (**e**) rotational viscosity.

Through conventional performance tests of asphalt, the penetration, softening point, ductility, segregation, and viscosity indicators of three types of asphalt were measured, with the test results shown in Figure 7.

Based on SBS-modified asphalt, the performance differences between ARA/SBS and RA/SBS can be summarized as follows. As shown in Figure 7a, the penetration of ARA/SBS is significantly higher than that of RA/SBS, indicating that activated rubber particles improve compatibility with asphalt. This enhanced compatibility stems from the increased polar groups on the activated rubber surfaces, promoting intermolecular interactions with asphalt phases. Figure 7b shows that ARA/SBS has a higher softening point than RA/SBS, attributed to the activated rubber particles strengthening the interfacial binding energy and suppressing high-temperature flow deformation [13]. Figure 7c reveals that activated rubber particles reduce asphalt ductility compared to RA/SBS, yet they exhibit superior performance since raw rubber powder has poor compatibility and inferior shearing effects compared to twin-screw activated rubber powder. Figure 7d demonstrates that incorporating activated rubber particles alters segregation, influenced by time, temperature, and primarily matrix asphalt properties and modifier characteristics/dosage [18]. A low rubber content yields minimal segregation, which increases with higher dosages until reaching a threshold and then decreases before rising again. Higher activation degrees improve rubber–asphalt compatibility, reducing segregation. Figure 7e shows that viscosity decreases with rising temperature across all asphalt types due to enhanced molecular thermal motion and weakened intermolecular forces. SBS (without activated rubber) exhibits the lowest viscosity due to its strong compatibility with matrix asphalt. Compared to RA/SBS, ARA/SBS demonstrates reduced viscosity via improved rubber-asphalt compatibility [10].

In summary, twin-screw activated rubber forms cross-linked networks with SBS, reducing system hardness (penetration). Activated rubber and SBS interpenetrate to create high-temperature frameworks, enhancing rutting resistance (softening point and ductility). Low-temperature crack resistance (ductility) decreases slightly compared to SBS due to partial agglomerates of activated rubber in asphalt, which remain unreacted and act as fracture initiation points [14,19]. However, ARA/SBS still outperforms RA/SBS, which contains more free agglomerates and fracture sites. Enhanced intermolecular forces from activation improve rotational viscosity, maintaining advantages over RA/SBS. Segregation is primarily influenced by storage duration, temperature, matrix asphalt properties, and modifier characteristics/dosage. Storage conditions follow standard specifications, while the asphalt asphaltene content and modifier dosage/properties positively correlate with segregation [7,11,20].

### 3.2. Dynamic Shear Rheological Test Analysis

(1) Temperature Sweep Test

Obtain the complex shear modulus G*, phase angle δ, and rutting factor G*/sinδ of three asphalt binders at different temperatures (52, 58, 64, 70, 76, and 82 °C) through temperature sweep tests using a DSR module and analyze their rheological properties. The complex modulus G* and phase angle δ of asphalt specimens can be calculated from shear stress S and shear strain D, as shown in Equations (1)–(4).

(1) $$S=\frac{2T}{\pi R^{3}}$$

(2) $$D=\frac{\theta \cdot r}{h}$$

(3) $$G^{*}=\frac{S_{\max}-S_{\min}}{D_{\max}-D_{\min}}$$

(4) δ=2πf⋅Δt

In the formula, T is the maximum torque; r is the radius of the oscillating plate (12.5 mm or 4 mm); h is the specimen height (1 mm or 2 mm); θ is the rotation angle of the oscillating plate; $S_{\max}$, $S_{\min}$, $D_{\max}$, $D_{\min}$ are the maximum/minimum shear stress and shear strain experienced by the specimen; and Δt is the hysteresis time.

**Figure 8:**
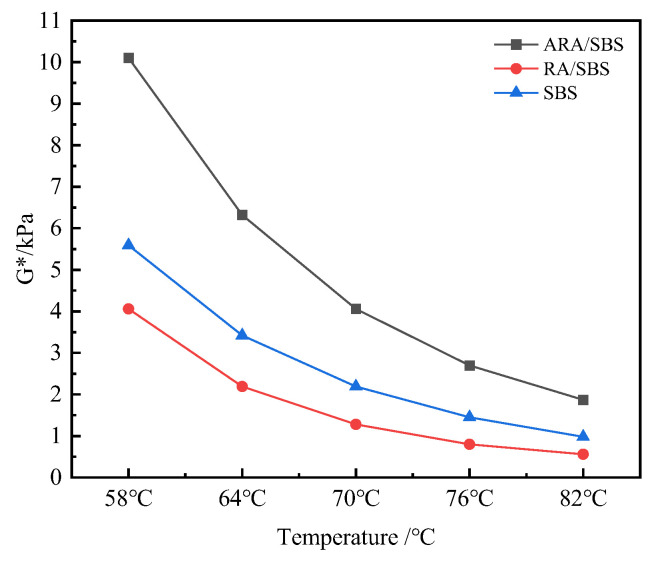
Complex shear modulus.

Through the analysis of G* using the aforementioned formula, Figure 8 shows that the G* of all three modified asphalts decreases with increasing temperature. A sharp decline occurs between 58 and 70 °C, followed by a significantly slower reduction trend from 70 to 82 °C, corresponding to the thermally activated migration mechanism dominated by saturates and aromatics in asphalt components. Under identical temperature conditions, the G* of ARA/SBS is notably higher than that of SBS and RA/SBS, indicating that activated rubber particles disperse more effectively in the asphalt phase, enhancing deformation resistance. This aligns closely with findings from Li Xiaojuan et al. [11,20]. At 82 °C, the G* of RA/SBS is 43% lower than that of SBS, while ARA/SBS exhibits a 91% increase compared to SBS. This confirms that twin-screw shear activation technology enhances phase dispersion by increasing polar functional groups on rubber powder surfaces, demonstrating the necessity of rubber activation. Furthermore, the lower G* of RA/SBS compared to SBS suggests stress concentration effects caused by the surface inertness of inactivated rubber powder. In contrast, the higher G* of ARA/SBS reflects the improved high-temperature deformation resistance through a “rigid–flexible synergistic” structure formed by activated rubber powder.

**Figure 9:**
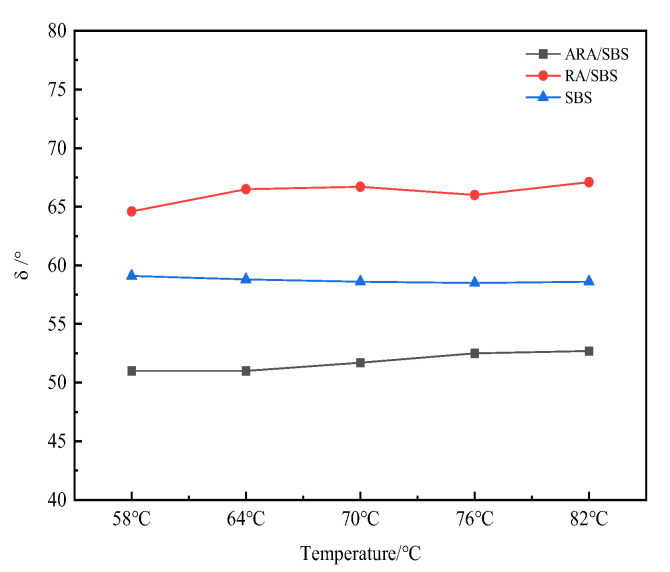
The phase angle.

The smaller the δ, the more the material tends to be elastic and the stronger its deformation resistance. According to the phase angle δ test results in Figure 9, the δ value of ARA/SBS is significantly lower than those of SBS and RA/SBS. This phenomenon is attributed to the chemical coupling interactions between carboxyl/hydroxyl groups on activated rubber particle surfaces and asphalt colloids, forming a three-dimensional cross-linked network structure that demonstrates remarkable elastic properties [16,21]. The δ value of SBS shows negligible variation with increasing temperature, indicating a more stable viscoelastic equilibrium derived from the dynamically maintained entanglement points of SBS block copolymer molecular chains within 58–82 °C. The fluctuating δ curve of RA/SBS reveals inhomogeneous dispersion between non-activated rubber particles and asphalt matrix, where aggregated non-activated rubber powders cause interface stress concentration and increased energy dissipation.

Rutting factor serves as the critical indicator for evaluating asphalt’s high-temperature rutting resistance, with higher values indicating better permanent deformation resistance. The rutting factor can be calculated using Equation (5).

(5) $$\tan\theta =G^{*}/\sin\theta$$

In the equation, $G^{*}$ is the complex shear modulus and θ is the rotation angle of the oscillating plate.

**Figure 10:**
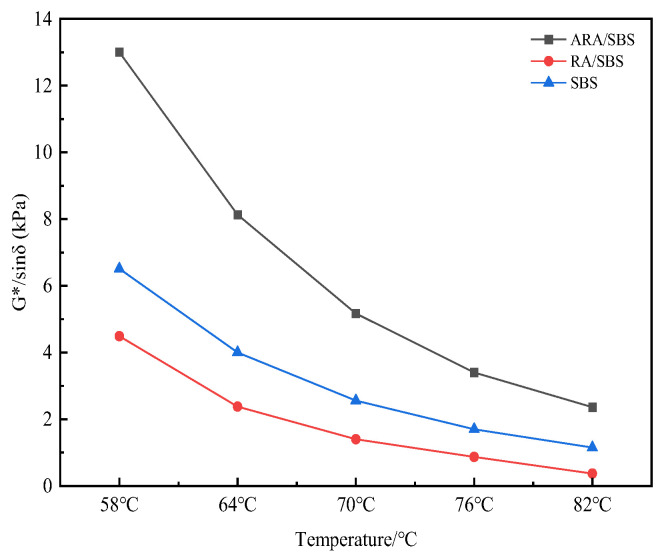
Rutting factor.

From the G*/sinδ test results in Figure 10, it can be observed that the G*/sinδ values of ARA/SBS, SBS, and RA/SBS all exhibit exponential decay as the temperature decreases. Although the decay rate of ARA/SBS is significantly higher than those of SBS and RA/SBS, the G*/sinδ of ARA/SBS remains greater than those of SBS and RA/SBS at the same temperature. At 82 °C, the G*/sinδ of ARA/SBS is 105% higher than that of SBS, while RA/SBS is 210% lower than SBS. This strongly confirms that the elastic network formed by the interaction between activated rubber powder and SBS enhances resistance to flow deformation. It fully demonstrates that the three-dimensional cross-linked network of rubber powder after twin-screw activation maintains structural integrity at high temperatures [22], significantly improving the anti-rutting capability of ARA/SBS under high-temperature conditions.

(2) Multi-Stress Creep Recovery Test

Since the rutting factor cannot comprehensively reflect the damage resistance level of composite-modified asphalt, the MSCR test was employed, still conducted on a DSR using a 25 mm rotor. The test simulated stress conditions of asphalt pavement under light (0.1 kPa) and heavy (3.2 kPa) traffic loads, with 10 cycles per stress level. Each cycle consisted of a 1 s creep phase and a 9 s unloading recovery phase, totaling 200 s. This yielded the creep recovery rate (R) and non-recoverable creep compliance (J_nr_), calculated via Equations (6) and (7). R reflects the elastic recovery capability (higher values indicate stronger recovery), while Jnr characterizes the resistance to permanent deformation (lower values indicate better performance). These parameters evaluate asphalt performance under different stress levels, with test data at 64 °C selected for comparative analysis in this study.

(6) $$R=\frac{\varepsilon_{p}-\varepsilon_{u}}{\varepsilon_{p}}\times 100\%$$

(7) $$J_{nr}=\frac{\varepsilon_{u}}{\sigma }$$

where $\varepsilon_{p}$ is the peak strain, $\varepsilon_{u}$ is the unrecovered strain, and σ is the stress.

**Figure 11:**
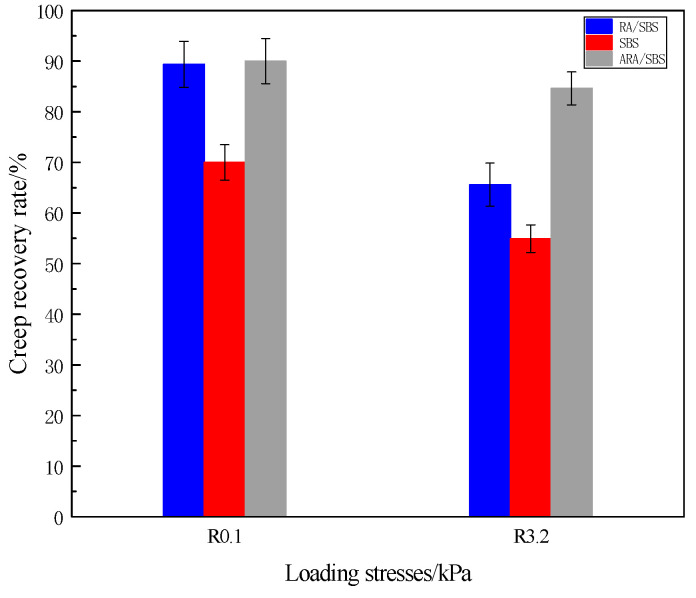
Creep recovery rate.

As shown in Figure 11, under the 0.1 kPa reference load, the R-values of ARA/SBS and RA/SBS are similar and both higher than SBS, indicating that the incorporation of rubber powder enhances asphalt’s recovery performance. When the load increases to 3.2 kPa, significant differences emerge among the three: ARA/SBS demonstrates superior stress adaptability with notably lower performance degradation compared to SBS and RA/SBS. The R-value represents asphalt’s ability to recover deformation after unloading under specific stress levels [23]. The Figure 11 results (ARA/SBS > RA/SBS > SBS) clearly show that under heavy traffic conditions, ARA/SBS modified asphalt pavement exhibits 30% better performance than SBS. This further proves that rubber powder activated through twin-screw technology can effectively improve the road performance of asphalt systems.

**Figure 12:**
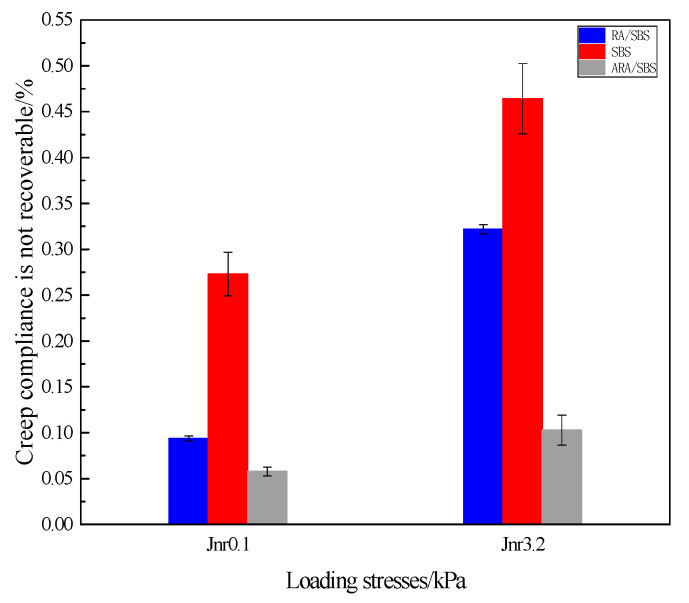
Creep compliance is not recoverable.

The non-recoverable creep compliance, Jnr reflects the degree of permanent deformation in asphalt under stress, with a higher applied stress resulting in greater Jnr values. According to the Jnr characterization results from the MSCR test in Figure 12, under the baseline stress level of 0.1 kPa, the order of non-recoverable creep compliance for the three materials is SBS > RA/SBS > ARA/SBS, indicating that the incorporation of activated rubber powder enhances resistance to permanent deformation. Among them, ARA/SBS demonstrates optimal rutting resistance due to solid-phase interface reconstruction effects. When the applied stress increases to 3.2 kPa, this ranking remains unchanged, verifying the stress-dependent characteristics of the Jnr parameter in the activated rubber powder-modified asphalt system. Additionally, ARA/SBS exhibits the lowest incremental rate, suggesting more stable mechanical performance under varying traffic load conditions. Ying et al. investigated the high- and low-temperature rheological properties of activated rubber powder-modified asphalt, and their conclusions align with this study [13,14,15].

(3) BBR Test Results Analysis

**Figure 13:**
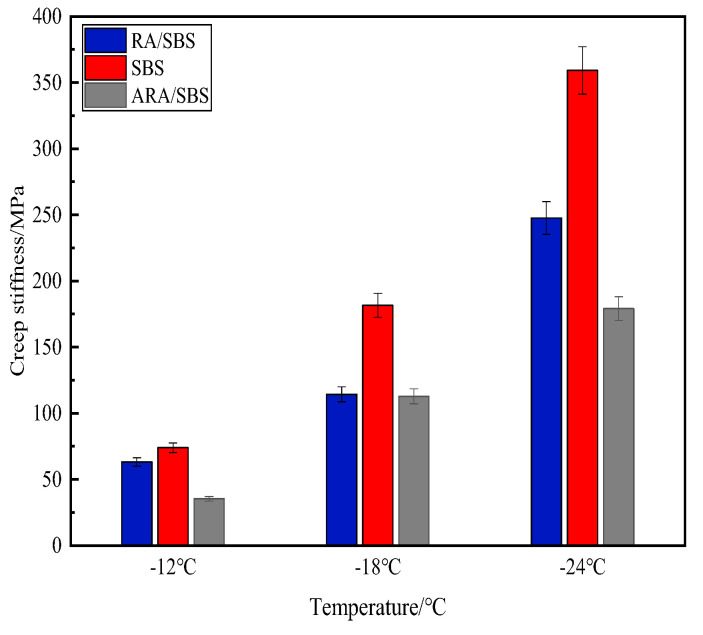
Creep stiffness.

**Figure 14:**
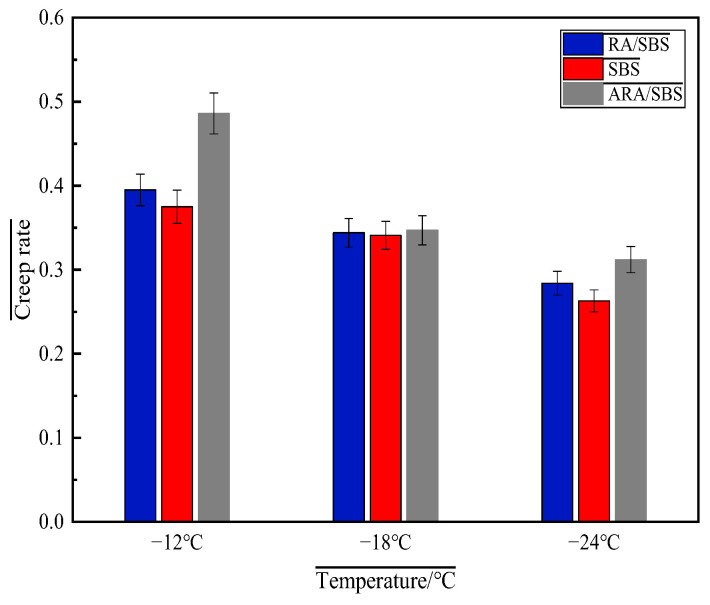
Creep rate.

This paper evaluates the low-temperature crack resistance of asphalt through BBR tests, where a smaller creep stiffness S and larger creep rate m indicate better low-temperature performance, with the test results shown in Figure 13 and Figure 14.

Based on the analysis of the low-temperature rheological properties from the BBR test in Figure 13, the creep stiffness S gradually increases with decreasing temperature. At −12 °C, −18 °C, and −24 °C, ARA/SBS exhibits the smallest creep stiffness, followed by RA/SBS, while SBS shows the largest value. Lower creep stiffness S indicates better low-temperature performance [21]. As temperature decreases, the S values of all three modified asphalts increase significantly, with SBS demonstrating the steepest growth trend, RA/SBS following, and ARA/SBS showing the smallest increment. Notably, the ARA/SBS system maintains the lowest S values across all tested temperatures. This difference originates from the energy dissipation network formed through the topological entanglement of flexible macromolecular chains in activated rubber powder [24], which remarkably improves low-temperature brittleness. However, a sole reliance on S values has limitations, necessitating further interpretation using m-values. Figure 14 reveals that the m-values of all three modified asphalts decrease with temperature reduction. Although ARA/SBS shows the most pronounced downward trend, it still maintains the highest m-values, with the ranking at −12 °C, −18 °C, and −24 °C being ARA/SBS > RA/SBS > SBS. This further confirms ARA/SBS’s superior low-temperature performance, followed by RA/SBS, while SBS performs the worst—consistent with the creep stiffness S analysis results.

### 3.3. FTIR Test Analysis

**Figure 15:**
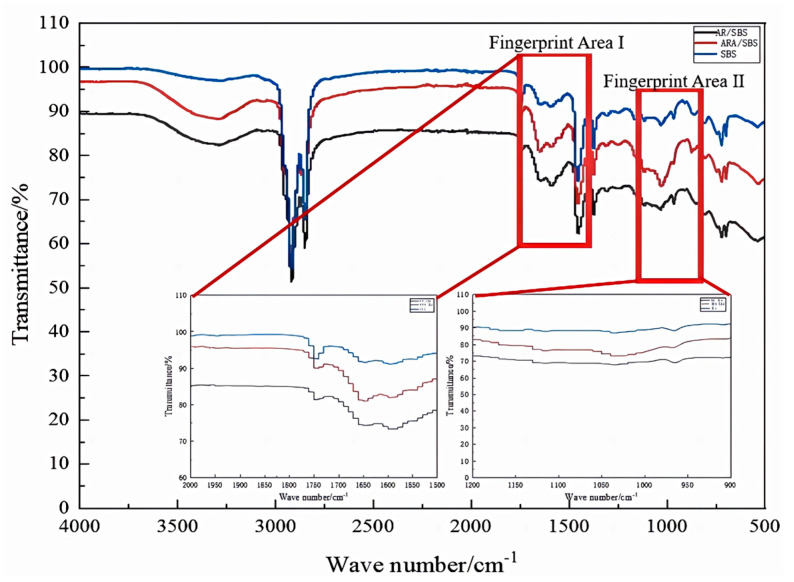
Infrared spectroscopy.

The characteristic functional groups of the three modified asphalt samples were analyzed using a Thermo Nicolet infrared spectrometer. The quality of the modification process was evaluated by examining the polymer particle size and distribution uniformity (e.g., whether SBS and rubber powder were uniformly dispersed). The key fingerprint regions were marked in the spectra, and the characteristic absorption peaks obtained are shown in Figure 15.

Based on the Fourier transform infrared spectroscopy (FTIR) characterization results (Figure 15), typical characteristic absorption bands appeared at 1720 cm^−1^ (fingerprint region I) and 1030 cm^−1^ (fingerprint region II). From the fingerprint area I magnification, ARA/SBS can be seen in the vicinity of 1720 cm^−1^, showing more obvious absorption peaks, corresponding to the carbonyl (C=O) of the tensile vibration; mainly asphalt “C=C” is oxidized [22], indicating that the rubber blending makes the modified asphalt partially oxidize, resulting in a reduction in the ductility of the modified asphalt. This is consistent with the results of the ductility test in Figure 7c. From the fingerprint area II magnification it can be seen that in the vicinity of 1030 cm^−1^, more obvious absorption peaks appear, corresponding to the S=O bond bending vibration; the main reason for this is that the rubber blending changed the modified asphalt immiscible system and could better promote the dissolution of SBS in the asphalt, so that the modified asphalt system was more stable. The microcosmic performance of the modification process of ARA/SBS is mainly based on Physicochemical modification [23], and the macrocosmic performance shows that the conventional performance indexes are more prominent than those of RA/SBS and SBS.

The functional group index was used to evaluate the effect of the solubility of desulfurized rubber powder on the characteristic peaks, and the calculation of the functional group index of the characteristic peaks was shown in Equations (8)–(10).

(8) $$I_{1720}=AR_{1720}/\Sigma AR_{500-2000}$$

(9) $$I_{1030}=AR_{1030}/\Sigma AR_{500-2000}$$

(10) $$\Sigma I=I_{1720}+I_{1030}$$

Among them, $I_{1720}$ and $I_{1030}$ has functional group indices at 1720 cm^−1^ and 1030 cm^−1^, respectively, and $AR_{1720}$ and $AR_{1030}$ peak areas at 1720 cm^−1^ and 1030 cm^−1^, respectively; $\Sigma AR_{500-2000}$ is the sum of the areas of all characteristic peaks in the range of 500 to 2000 cm^−1^, and ΣI is the sum of the functional group indices of the characteristic peaks of the bitumen.

Table 5 shows the calculation results of the main characteristic peak areas and functional group indexes of different desulfurization rubber powders.

As can be seen from Table 5, the carbonyl and sulfenyl indices of ARA/SBS were enhanced by 5.5% and 9.0% compared with SBS and RA/SBS systems, respectively. The particular reason is that the high thermo-mechanical energy of the twin-screw triggers the C-S/S-S bond breakage of the gum powder, releasing the activated radicals and sulfur atoms and promoting the sulfenyl group reconstruction. In addition, the activated system shows the symmetric equilibrium characteristics of a carbonyl/sulfinyl group, while the lag of sulfinyl group increment in the RA/SBS system and the limitation of the low sulfur content in SBS confirm that sulfur migration is the key to the construction of a cross-linked network. It is fully demonstrated that the bifunctional group modulation constructs a dual anti-rutting barrier—the carbonyl group polarity enhances the hydrogen bonding effect, and the sulfenyl group cross-linking accelerates the three-dimensional network reconstruction.

In summary, FTIR reveals the interfacial evolution of adhesive powder activation through chemical bonding vibration characteristics; the dynamic shear rheological test quantifies the high-temperature rheological response through temperature-frequency scanning and finds that the adhesive powder effectively suppresses phase separation and enhances the strength of the elastic network after twin-screw activation treatment. The bending-beam rheological test focuses on low-temperature rutting resistance, which proves that the formation of interfacial chemical bonds in the twin-screw activation system alleviates the rigidity of the powder and the low-temperature stress concentration of the adhesive powder. It proves that the formation of interfacial chemical bonds in the twin-screw activation system alleviated the low-temperature stress concentration effect of the adhesive powder rigid aggregate. Comprehensively, the twin-screw activation process regulates the interfacial microstructure through chemical bonding and optimizes the high- and low-temperature rheological response of the rubber powder–asphalt composite system simultaneously.

### 3.4. Fluorescent Microscope Test Analysis

**Figure 16:**
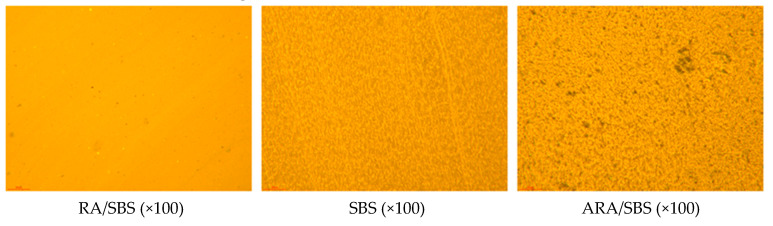
Rubber composite modified asphalt production process.

Fluorescence microscopy tests were conducted on three types of modified asphalt to obtain fluorescence images. The matrix asphalt (non-fluorescent phase) served as the continuous phase. The phase structure of asphalt was analyzed, focusing on the shape, size, and uniformity of the activated rubber particle distribution, and on the continuity between asphalt and rubber phases. The phase structure of modified asphalt largely determines its thermal storage stability and other properties. Fluorescence micrographs are shown in Figure 16.

In RA/SBS, the raw rubber powder is fully and uniformly mixed with the asphalt, with almost no accumulation clusters of rubber powder. The individual rubber particles are distributed relatively evenly, with single rubber particles tightly integrated with the dispersed phase in the asphalt colloidal structure through dissolution into the asphalt or partial swelling followed by dissolution, forming a relatively stable system that maximizes the viscosity of the modified asphalt. In SBS, the distribution state of SBS within the asphalt can be clearly observed, with SBS and asphalt interpenetrating each other. This significantly enhances asphalt performance, particularly the ductility and softening point. SBS particles interconnect with asphalt particles, forming a cross-linked network structure [25], greatly improving the storage stability of modified asphalt. In ARA/SBS, most activated rubber surfaces have been dissolved by light components in the asphalt, with a small amount of light components penetrating into the activated rubber particles. This occurs because under high-temperature conditions, activated rubber particles absorb certain components from the asphalt after thorough mixing, leading to dissolution and swelling phenomena while forming a gel film on their surfaces [25]. Additionally, the volume expansion of swollen activated rubber particles and the gel film enhance interparticle connectivity, increasing asphalt viscosity. The activated rubber particles fill the asphalt to form a skeletal structure, further demonstrating their filling effect in enhancing asphalt’s road performance.

## 4. Conclusions

(1) Through the above experimental characterization, it can be seen that ARA/SBS composite-modified asphalt shows a significant multidimensional performance synergistic effect, which confirms that the activated rubber powder can give the material excellent viscoelastic properties, improve the storage stability, and also show good high temperature performance.
(2) High activation degree of rubber particles on the asphalt system modification presents two-sided effect: on the one hand, the interface compatibility enhancement can inhibit the tendency for phase separation, but excessive activation will trigger local agglomeration due to insufficient participation in the matrix asphalt cross-linking reaction in the dynamic load, evolving into a crack-sprouting point, resulting in a decrease in ductility; on the other hand, the higher the activation degree, the better the compatibility of rubber and asphalt, so the softening point and the degree of needle entry increase; thus, the dosage of activated rubber particles must be strictly controlled.
(3) RA/SBS will trigger the stress concentration effect due to surface inertia, making the asphalt performance enhancement limited; twin-screw shear activation technology can be realized to enhance the phase dispersion effect by increasing the surface of the rubber powder polar functional groups, while encouraging coupling between the carboxyl and hydroxyl groups in the ARA/SBS and asphalt gel, causing the chemical formation of a three-dimensional cross-linked network structure. The constructed structure greatly improved the high- and low-temperature capability of asphalt.
(4) ARA/SBS showed the emergence of obvious functional groups at 1750 cm^−1^ and 1075 cm^−1^ due to the surface oxidation of functional groups and asphalt components in the polar aromatic structure of the reaction. This shows that the activation of the rubber particles and matrix asphalt matches the degree of enhancement, prompting the composite-modified asphalt system to show enhanced interfacial compatibility, and ultimately showing the synergistic optimization of the performance indexes.
(5) The twin-screw extruder activation significantly improves the reaction activity of the rubber powder, activated rubber powder, and asphalt/SBS combination; in detail, the asphalt rutting factor increases and both the high-temperature rutting and low-temperature cracking resistance show optimization, compared with the traditional process used to enhance the mixing of the rubber powder. The process also promotes the use of waste tires resources, providing a comprehensive cost reduction.
(6) The industrialization of the twin-screw activation process can significantly reduce energy consumption, enhance the efficiency of waste rubber regeneration through continuous production, and reduce the accumulation of solid waste and carbon emissions. In terms of economic benefits, the integration of equipment can improve the production capacity, promote growth in the product interface bonding strength, and is applicable to high-value areas such as highway engineering, airport runways, etc. Additionally, green recycling, profitability, and efficiency metrics are all improved.

## Figures and Tables

**Table 1:** Zhenhai 90# matrix asphalt general properties.

Performance Indicators	Zhenhai 90#
Penetration of a needle (25 °C, 100 g, 5 s)/0.1 mm	89.2
Softening point (International Law)/°C	44.0
Ductility (5 cm/min, 10 °C)/cm	150.19
135 °C Rotational viscosity/pa·s	0.503
25 °C Elastic recovery/%	12
Residues after aging in rotary film ovens (163 °C, 85 min)	
Mass loss/%	0.1301
Needle penetration ratio/%	62
Ductility (5 cm/min, 10 °C)/cm	44.9

**Table 2:** Physical and chemical technical specifications of 40-mesh gum powder.

Sports Event	Sieve Residue (%)	Relative Density (g/cm^3^)	Moisture Content (%)	Metal Content (%)	Fiber Content (%)
Technical indicators	<10	1.10~1.30	<1	<0.03	<1
**Sports Event**	**Ash (%)**	**Acetone Extract (%)**	**Carbon Black Content (%)**	**Rubber Hydrocarbon Content (%)**	
Technical indicators	≤8	≤16	≥28	≥48	

**Table 3:** The main properties of furfural extracted oil.

Sports Event	Numbers
Densities (20 °C)/(g·cm^3^)	0.9895
Index of refraction	1.5610
Flash point (open)/°C	270
Relative average molecular weight	357

**Table 4:** Stabilizer quality indicators.

Quality Indicators	Numbers
Apparent density/g·cm^3^	0.8
Melting point	≥110 °C
Fineness (80 mesh sieve residue)	≤2%
Hydration	≤0.5%

**Table 5:** FTIR peak area and functional group index calculation results.

**Type of Asphalt**	$\Sigma AR_{500-2000}$	$AR_{1720}$	$AR_{1030}$	$I_{1720}$	$I_{1030}$	ΣI
SBS	2167.7	108	376.7	0.050	0.174	0.674
RA/SBS	2771.6	702	678	0.253	0.245	0.498
ARA/SBS	3630.6	968	1150.9	0.267	0.317	0.584

## Data Availability

The original contributions presented in this study are included in the article. Further inquiries can be directed to the corresponding authors.
